# Imaging temozolomide-induced changes in the myeloid glioma microenvironment

**DOI:** 10.7150/thno.47269

**Published:** 2021-01-01

**Authors:** Claudia Foray, Silvia Valtorta, Cristina Barca, Alexandra Winkeler, Wolfgang Roll, Michael Müther, Stefan Wagner, Miranda L. Gardner, Sven Hermann, Michael Schäfers, Oliver Martin Grauer, Rosa Maria Moresco, Bastian Zinnhardt, Andreas H. Jacobs

**Affiliations:** 1 European Institute for Molecular Imaging - EIMI, University of Münster, Münster, Germany.; 2 PET Imaging in Drug Design and Development (PET3D), Münster, Germany.; 3 Tecnomed Foundation and Medicine and Surgery Department, University of Milano-Bicocca, Milan, Italy.; 4 Experimental Imaging Center, IRCCS San Raffaele Scientific Institute, Milan, Italy.; 5 Institute of Molecular Bioimaging and Physiology (IBFM), CNR, Segrate (MI), Italy.; 6 Université Paris-Saclay, CEA, CNRS, Inserm, BioMaps, Orsay, France.; 7 Department of Nuclear Medicine, University Hospital Münster, Münster, Germany.; 8 Department of Neurosurgery, University Hospital Münster, Münster, Germany.; 9 Campus Chemical Instrument Center, Mass Spectrometry and Proteomics Facility (CCIC-MSP), The Ohio State University, Columbus, OH, United States.; 10 Cells in Motion (CiM) Cluster of Excellence, Münster, Germany.; 11 Department of Neurology, University Hospital Münster, Münster, Germany.; 12 Imaging Neuroinflammation in Neurodegenerative Diseases (INMIND) EU FP7 consortium, Münster, Germany.; 13 Department of Geriatrics, Johanniter Hospital, Evangelische Kliniken, Bonn, Germany.

**Keywords:** glioblastoma, temozolomide, GAMM, [^18^F]DPA-714, [^18^F]FET, TSPO

## Abstract

**Rationale:** The heterogeneous nature of gliomas makes the development and application of novel treatments challenging. In particular, infiltrating myeloid cells play a role in tumor progression and therapy resistance. Hence, a detailed understanding of the dynamic interplay of tumor cells and immune cells *in vivo* is necessary. To investigate the complex interaction between tumor progression and therapy-induced changes in the myeloid immune component of the tumor microenvironment, we used a combination of [^18^F]FET (amino acid metabolism) and [^18^F]DPA-714 (TSPO, GAMMs, tumor cells, astrocytes, endothelial cells) PET/MRI together with immune-phenotyping. The aim of the study was to monitor temozolomide (TMZ) treatment response and therapy-induced changes in the inflammatory tumor microenvironment (TME).

**Methods:** Eighteen NMRI^nu/nu^ mice orthotopically implanted with Gli36dEGFR cells underwent MRI and PET/CT scans before and after treatment with TMZ or DMSO (vehicle). Tumor-to-background (striatum) uptake ratios were calculated and areas of unique tracer uptake (FET vs. DPA) were determined using an atlas-based volumetric approach.

**Results:** TMZ therapy significantly modified the spatial distribution and uptake of both tracers. [^18^F]FET uptake was significantly reduced after therapy (-53 ± 84%) accompanied by a significant decrease of tumor volume (-17 ± 6%). In contrast, a significant increase (61 ± 33%) of [^18^F]DPA-714 uptake was detected by TSPO imaging in specific areas of the tumor. Immunohistochemistry (IHC) validated the reduction in tumor volumes and further revealed the presence of reactive TSPO-expressing glioma-associated microglia/macrophages (GAMMs) in the TME.

**Conclusion:** We confirm the efficiency of [^18^F]FET-PET for monitoring TMZ-treatment response and demonstrate that *in vivo* TSPO-PET performed with [^18^F]DPA-714 can be used to identify specific reactive areas of myeloid cell infiltration in the TME.

## Introduction

The most frequently diagnosed primary tumor in the central nervous system is glioma, further classified as astrocytoma, oligodendroglioma, or ependymoma depending on histological and genetic features 1.

The most prevalent glioma, glioblastoma multiforme (GBM), is highly heterogeneous in nature. GBM tumors are comprised of multiple cells of origin and differentiation, from stem cells and the peripheral immune system to tumor-associated parenchymal cells, such as vascular endothelial cells, pericytes, microglia and tumor precursor cells 2. One diverse population of myeloid-derived cells among them, glioma-associated microglia/macrophages (GAMMs), play a fundamental role in tumor growth, progression and therapy resistance 3,4. Through secretion of cytokines and growth factors, such as IL-4, CCL2, CSF1 and TGF-β, GAMMs sustain the tumor proliferation and the evasion of immune surveillance 5,6. Given this information, multiple novel treatments targeting the myeloid cell compartment are currently under investigation 4,7.

Current therapy options for GBM are limited to surgical resection, followed by radiotherapy and concomitant/adjuvant chemotherapy with alkylating agents such as temozolomide (TMZ) 8. However, these therapeutic options are severely limited as the median overall survival is typically 14-15 months 9. The efficacy of TMZ therapy depends on the expression levels of the DNA-repair protein MGMT (O^6^-methylguanine-DNA methyltransferase), responsible for the removal of DNA methyl adducts which cause DNA damage. When MGMT is inhibited, TMZ therapy is more effective; on the other hand, if the DNA repair activity of this protein is intact, the cells acquire resistance to TMZ 10. One additional challenge includes monitoring therapy outcomes due to the complex heterogeneity of the tumor tissue and the location where the tumor develops inside the brain that influence therapy resistance and sensitivity 11,12. For example, MRI alone cannot discriminate between tumor progression and pseudoprogression. Additionally, MRI results are negatively influenced by therapeutic effects, such as pseudoresponse occurring during VEGFR therapy 12. Molecular imaging can complement the use of MRI 13 and be of help to better identify specific non-responsive tumor areas. For this reason, combinatorial approaches using non-invasive imaging, such as amino acid PET, with MRI have become the guideline-recommended standard to diagnosis and classification of gliomas 14.

Dedicated imaging tools with the capacity to simultaneously monitor the myeloid cell compartment, as well as dynamic changes in the TME in response to routine clinical therapies are lacking. To address this problem, different radiotracers targeting the myeloid cell compartment have been developed 15,16. Among them, ligands targeting the 18kDa translocator protein (TSPO) have major relevance 17. TSPO is expressed in glioma tissues mostly by tumor cells, GAMMs and endothelial cells 18-20.

TSPO in gliomas can be non-invasively assessed with the second generation PET tracer, *N,N*-diethyl-2-(2-(4-(2-[^18^F]fluoroethoxy)phenyl)-5,7-dimethylpyrazolo[1,5-a]pyrimidin-3-yl)acetamide([^18^F]DPA-714) 21.

Recent studies of TSPO-PET in conjunction with in-depth immunophenotyping suggested [^18^F]DPA-714 to non-invasively image the degree and heterogeneity of the glioma-associated immunosuppressive tumor microenvironment (TME) *in vivo* 22,23. In addition, [^18^F]DPA-714 may reveal distinct areas of glioma activity 22,23 compared to other imaging biomarkers and may be superior over [^18^F]FET in detection of glioma infiltration 24.

Here, a multi-tracer- and multimodal-imaging approach is used to investigate the ability to image therapy response and neuro-inflammation in relation to tumor growth and tumor microenvironment in a mouse model of human orthotopic glioma. We assessed the value of an approach combining [^18^F]FET (amino acid metabolism) with [^18^F]DPA-714 PET (TSPO, GAMMs) and MRI (i) to investigate non-invasively tumor growth and tumor-associated inflammation, (ii) to monitor temozolomide (TMZ) treatment response *in vivo,* and (iii) to analyze possible therapy-induced reactive changes in the TME.

We hypothesized that [^18^F]DPA-714 tracer uptake is influenced by reactive TMZ-induced myeloid cell infiltration into the TME and that [^18^F]DPA-714-PET can be used as imaging biomarker for the assessment of TMZ efficacy, as well as reactive myeloid cell infiltration and activation.

## Materials and methods

### Study design

The time points and time frames outlined below were chosen based on clinical imaging protocols and preliminary imaging results, monitoring in particular the tumor progression in control animals 22,23.

Female NMRI^nu/nu^ mice (Janvier, France), 8-12 weeks old, were housed at constant temperature and relative humidity under a regular light/dark schedule. Food and water were available *ad libitum*.

In total, 20 = mice were orthotopically implanted (intra-striatal injection, coordinates in relation to bregma: lateral -2.0 mm, anterior-posterior +0.5 mm, dorsal-ventral -3.0 mm) with 2 × 10^5^ human Gli36dEGFR-LITG cells in 2 µl NaCl 0.9%. During anaesthesia, body temperature was maintained at physiological level with a custom-built heating pad. Ten days post implantation (p.i.) mice were treated daily with TMZ (50 mg/kg; Sigma Aldrich) or DMSO as vehicle control for 5 days.

Eighteen mice (n = 6 DMSO; n = 12 TMZ) were successfully imaged at different time points, pre- and post-therapy. Two mice could not be analyzed due to failure in the tracer synthesis. Eleven mice underwent surgery without imaging and five of them did not receive any treatment, constituting the control group. First, T2-weighted (T2w) MRI and T1-weighted (T1w) MRI, native and with gadolinium as contrast agent, were performed at day 10 post-implantation (p.i.), followed by PET acquisition with [^18^F]FET on the same day, and [^18^F]DPA-714 on the next day. The same acquisitions were performed again 17 (T2w MRI, T1w CE-MRI and [^18^F]FET-PET) and 18 days ([^18^F]DPA-714 PET) p.i.

All experiments were conducted in accordance with the German Law on the Care and Use of Laboratory Animals and approved by the Landesamt für Natur, Umwelt und Verbraucherschutz (LANUV) of North Rhine-Westphalia and the ARRIVE guidelines 25.

### Cell culture

Gli36dEGFR glioma cells were obtained from Dr. David Louis (Molecular Neurooncology Laboratory, Massachusetts General Hospital, Boston, MA) 26,27. The cells were engineered to express the luciferase reporter system (Gli36dEGFR-LITG) as previously described 28. Glioma cells were carefully cultured and observed. Tumor cells displayed the typical growth patterns and phenotypes *in vitro* and *in vivo*. The cells were not further genetically authenticated. Thawed cells were cultured in T-75 cell culture flasks (Greiner Bio One, Germany) as adherent monolayer in DMEM (life Technologies) supplemented with GlutaMax, 10% heat inactivated FCS (Invitrogen) and 1% penicillin/streptomycin (PAA Laboratories) at 37°C in a humidified incubator maintained at 5% CO_2_ prior to intracranial implantation.

### MRI studies

MRI was conducted for identification of glioma location, and for co-registration with PET/CT images. The mice were anaesthetized and the lateral tail vein was cannulated using a 26 Ga catheter (Vasculon Plus, BD, Heidelberg, Germany). A T2 FSE 2D sequence and a T1 SE 2D sequence were acquired in a 1T nanoScan PET/MRI scanner equipped with a MH20 coil (Mediso Medical Imaging Systems). MRI images have a final resolution of 0.27×0.27×0.9 mm. Gadovist (Bayer Vital GmbH, Leverkusen, Germany) was injected via the catheter (50 µmol/kg) and a post-Gd T1 sequence was acquired.

### PET studies

During all experimental procedures, mice were anesthetized with 1.5% to 2% isoflurane (Abbott Animal Health) in 100% O_2_. Mice were subjected to PET imaging using the radiotracers [^18^F]FET and [^18^F]DPA-714 for assessment of amino acid transport and TSPO expression, respectively. PET images were acquired on a high-resolution small animal PET scanner (32 module quadHIDAC, Oxford Positron Systems Ltd.) with uniform spatial resolution (<1 mm FWHM). PET data were 3D reconstructed using one-pass list mode expectation maximization algorithm with resolution recovery 29,30. Based on previous studies 22,23, [^18^F]DPA-714 PET images were acquired 60 to 80 minutes post-intravenous (i.v.) injection of 15.9 ± 3.3 MBq [^18^F]DPA-714. [^18^F]FET PET images were acquired 20 to 30 minutes post-i.v. injection of 10.4 ± 0.3 MBq of [^18^F]FET. After each PET acquisition, the animal bed was transferred into the computed tomography (CT) scanner (Inveon, Siemens Medical Solutions) for anatomic co-registration using a landmark based approach 31.

### Data analysis

Image data were analyzed using the in-house developed software MEDgical. Tumor volume, tumor-to-background (striatum) uptake ratios defined as (T_max_/B_max_ and T_mean_/B_mean_) and abbreviated as (T/B) moving forward and unique area of tracer uptake were calculated using an atlas-based volumetric approach. Volume-of-interest regions (VOI) defining the area of interest were placed using the brain atlas as reference, after co-registration of PET/CT with MRI. Two different VOI were defined: (i) a control VOI corresponding to the left striatum region (volume 11 mm³) and (ii) a VOI corresponding to the right hemisphere (volume 49 mm³), placed in the tumor-affected brain hemisphere, excluding the cerebellum. To avoid the inclusion of spill-in activity from the scalp into the analysis, the brain atlas and the VOIs were placed keeping a small distance from the skull. Data quantification was performed applying a thresholding approach, multiplying the standard deviation of the control VOI (striatum) by a factor of 3.5. The resulting counts were added to the mean values of the control region. This approach was used for both tracers and excluded the potential spill-in activity from submandibular and Harderian glands in the analysis. Tumor-to-background ratios (T/B) were calculated between the thresholded glioma VOI (mean and max values) and the contralateral control VOI (mean values). The co-registration, thresholding and volumetric analyses are represented in **Figure S1**.

### Immunohistochemistry and immunofluorescence

After the last imaging examination, mice were sacrificed and perfused with 0.9% NaCl and 4% PFA. Brains were fixed in 4% PFA O/N, embedded in paraffin and cut in 10 µm coronal sections. Immunohistochemistry was performed for all animals using the paraffin embedded coronal brain sections employing antibodies for microglia (1:250, rabbit anti Iba1, 019-19741, Wako), TSPO (1:250, rabbit anti PBR, ab109497, Abcam, Cambridge UK) and GFAP (1:500, chicken anti GFAP, ab13970, Abcam, Cambridge UK). Antigen retrieval was performed by boiling the slides in citrate buffer (pH 6; 18 min). Slides were then treated with blocking solution at RT for one hour (1% BSA and 0.5% Triton-X in PBS), subsequently incubated (4 °C O/N) with the primary antibodies, followed by incubation with Alexa Fluor 488-conjugated anti-rabbit secondary antibody (1:1000, A-21206, life technologies, Carlsbad, USA), or Alexa Fluor 555-conjugated anti-rabbit (1:1000, A-21432, life technologies, Carlsbad, USA). Nuclei were stained with DAPI (0.2 µg/ml in PBS, Roth, Karlsruhe, Germany). Slices were embedded using Mowiol solution (6 g glycerol, 2.4 g Mowiol 4-88 (0713, Roth, Karlsruhe, Germany), 6 ml distilled water, 12 ml Tris-HCl (pH8.5)). For conventional histology, slides were incubated with a biotinylated goat anti-rabbit (1:800, 45 min, B21078, Life Technologies, Darmstadt, Germany), followed by HRP-Streptavidin incubation (1:600, 20 min, K1016, Dako, Hamburg, Germany). The staining was visualized by incubation with 3,3 Diaminobenzidine (D-5637, Sigma, St. Louis, USA) for 5 min. Sections were counterstained with hematoxylin, dehydrated and mounted using Entellan (Merck, Darmstadt, Germany). Images were acquired with a combined fluorescence-light microscope (Nikon Eclipse NI-E, Nikon, Japan). Positive cells were quantified by manually counting the number of cells present in 20× magnification images taken from biological triplicates.

### Western blot

Tissue samples were collected, cut into left and right hemisphere and immediately frozen in liquid nitrogen. To perform protein extraction, the samples were homogenized in RIPA buffer (ab156034, Abcam, Cambridge, UK) supplemented with phosphatase inhibitor (PhosSTOP, Merck, Darmstadt, Germany) and protease inhibitor (PMSF, Merck, Darmstadt, Germany). The samples were diluted at a final concentration of 30 µg in 15 µl total volume, separated on SDS-polyacrylamide gels (Mini-PROTEAN TGX precast gel, Bio-Rad) and transferred to PVDF nitrocellulose membrane (Roti-PVDF, Roth, Karlsruhe, Germany). Membranes were blocked with either 3% BSA or 5% milk and incubated at 4 °C overnight with primary antibodies against Iba1 (rabbit 019-19741, Wako, Neuss, Germany), TSPO (rabbit anti PBR, ab109497, Abcam, Cambridge, UK) and GAPDH (ab37168, Abcam, Cambridge, UK). The secondary antibodies used were polyclonal goat anti-rabbit IgG/HRP (P0448, Dako, Hamburg, Germany) and polyclonal rabbit anti-mouse IgG/HRP (P0260, Dako, Hamburg, Germany). Densitometry analyses were performed by evaluating band intensity of mean grey value, followed by relative protein quantification with ImageJ software (National Institutes of Health, MD, USA) 32.

### Multiparametric flow cytometry (FACS)

Tumors and the correspondent contralateral sections were dissected from the brains of DMSO- and TMZ-treated animals without extracranial tumor growth (N = 6, n=3 per group), minced and incubated with Collagenase type IA (10 mg/ml, Merck, Darmstadt, Germany), DNase I (10 mg/ml, Merck, Darmstadt, Germany) and protease inhibitor (PMSF 1 mg/ml, Merck, Darmstadt, Germany) at 37 °C for 30 min. Tissues were further processed by pipetting up and down for 5 min and the cell suspensions were then filtered through a 40 µm cell strainer (Corning cell strainer, Merck, Darmstadt, Germany). Myeloid-derived cells were isolated performing a gradient centrifugation (75% Lymphoprep, Stemcell technologies, Vancouver, Canada), washed with FACS buffer (2% FCS in PBS) and stained with a panel of directly labeled monoclonal antibodies (mAbs). This antibody mixture included 7-AAD, PerCP/Cy5.5 anti-Ly6G, APC anti-CD11b, AlexaFluor 700 anti-CD45, APC/Fire 750 anti-Ly6C, Brilliant Violet 421 anti-CD68, Brilliant Violet 510 anti-IA/IE, FITC Isotope control antibody (all BioLegend, San Diego, California, USA) and AlexaFluor 488 recombinant anti-PBR (EPR5384, Abcam, Cambridge, UK). All samples were analyzed using the NaviosTM flow cytometer and the Kaluza 2.1 Software (Beckman Coulter, Krefeld, Germany). CD45^+^ cells were selected in a side scatter (SSC) vs CD45 plot. Viable cells were selected in a 7-AAD vs CD45 plot and then other subsets were defined: CD11b^+^Gr1^+^ (MDSCs), Ly6G^+^Ly6C^-^ (PMN-MDSCs), Ly6G^-^Ly6C^+^ (Mo-MDSCs). The expression of CD68, CD206 and MHC class II were tested as markers of activation and M2-like phenotype.

### Statistical analysis

All statistical analyses were performed using Prism 6 (GraphPad Software, Inc., USA). Differences over time in radiotracer uptake ratios and tracer uptake volumes intra- and inter-groups were tested using either one-way ANOVA with multiple comparisons corrected with Holm Sidak's test or a t-test, eventually followed by Mann-Whitney U test on ranks and Wilcoxon test, with Bonferroni correction for multiple measurements. Results are visualized with box plots depicting min, max and mean. Significance levels were set at *p* < 0.05. All results are shown as mean differences ± SE.

## Results

### [^18^F]FET- and [^18^F]DPA-714 PET/MRI allow monitoring therapy-response

**Figure 1:**
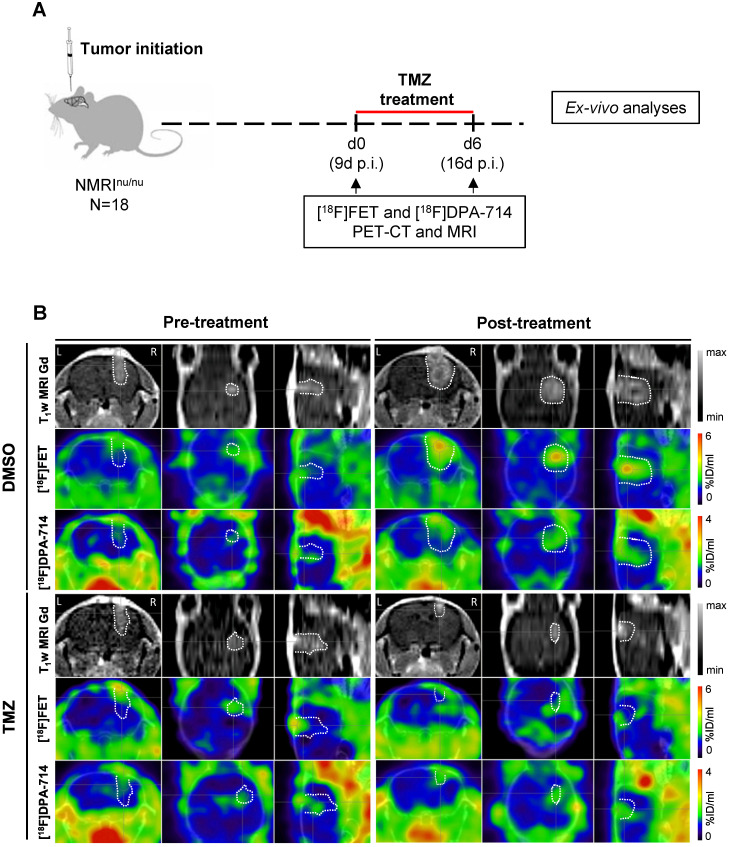
** Multimodal dual-tracer study showing the suitability of PET/MRI for monitoring temozolomide therapy response**. **(A)** Overview of the experimental workflow.** (B)** Representative T_1_wMRI Gd images and PET images for [^18^F]FET and [^18^F]DPA-714 (top to bottom) fused with CT of control (DMSO) and TMZ-treated animals, pre- and post-treatment (left to right). The dotted line indicates the tumor area depicted by MRI and transferred to PET images. L and R indicate left- and right-hemisphere. DMSO: dimethyl sulfoxide; TMZ: temozolomide.

Pre- and post-treatment scans were acquired for NMRI^nu/nu^ mice orthotopically implanted with human glioma cells (Gli36dEGFR-LITG) and treated with either vehicle (DMSO) or TMZ (**Figure 1A**). Gadolinium contrast-enhanced (CE) T1w MRI indicated increasing tumor volumes in DMSO-treated animals and a reduction of tumor volumes after TMZ therapy (**Figure 1B and Figure S2 - T1w MRI Gd**). Volumetric analyses of the gadolinium-enhanced MR images showed a significant increase in tumor volume in the DMSO-treated animals (0.023 ± 0.004 cm^3^; *p* ≤ 0.0001), whereas no modifications were observed in TMZ treated animals. Further comparison of the tumor volumes at day 6 post-treatment, showed a significant decrease between TMZ- and DMSO-treated animals (0.021 ± 0.003 cm^3^; *p* ≤ 0.0001) (**Figure S3A**).

Compared to the DMSO-treated group, TMZ treatment decreased [^18^F]FET uptake as indicated by [^18^F]FET-PET (**Figure 1B and Figure S2 - [^18^F]FET**). Alternative PET tracer [^18^F]DPA-714 was detected throughout the experiment, before and after therapy. Specifically, prior to therapy, the uptake was detectable at the site of tumor implantation, as well as at the border of the tumor mass. After one week of treatment, the [^18^F]DPA-714 signal matched the hot spot area of the tumor detected by [^18^F]FET-PET, and localizing also at the border of the neoplasm in DMSO-treated mice. Conversely, in the TMZ-treated group, the tracer uptake was primarily concentrated within the tumor area itself (**Figure 1B and Figure S2 - [^18^F]DPA-714**).

**Figure 2:**
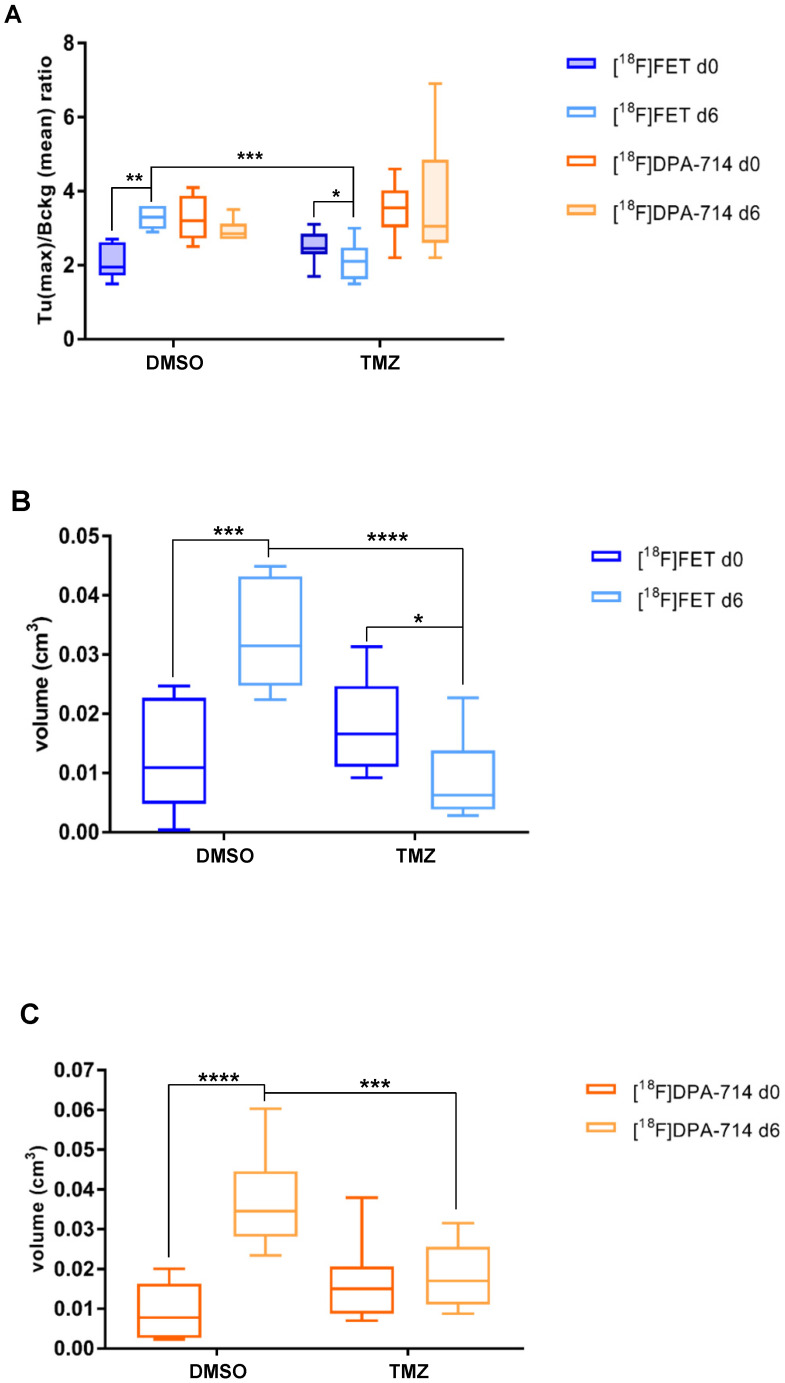
** [^18^F]FET- and [^18^F]DPA-714 PET detect tumor volume changes induced by TMZ therapy. (A)** Quantitative analysis of [^18^F]FET and [^18^F]DPA-714 T (max)/B (mean) uptake ratio after 6 days from the beginning of the therapy with DMSO (vehicle) and 50 mg/kg TMZ. **(B)** Volumetric analysis of [^18^F]FET and **(C)** [^18^F]DPA-714 uptake within the tumor region, pre- and post-therapy. Differences intra- and inter-groups were tested for significance using one-way ANOVA with multiple comparisons corrected for Holm-Sidak´s test. DMSO: dimethyl sulfoxide; TMZ: temozolomide

T/B analyses demonstrated a significant increase in [^18^F]FET signal in DMSO-treated mice (1.2 ± 0.4; *p*≤0.01). TMZ treatment resulted in significant reduction of T/B ratios (0.35 ± 0.30; *p* ≤0.05). At day 6, the [^18^F]FET signal between the two groups was decreased (1.15 ± 0.37; *p* ≤ 0.001) (**Figure 2A**). Similarly, mean T/B ratios followed the same trend: increased [^18^F]FET uptake in the DMSO-treated group (0.3 ± 0.15; *p* ≤ 0.05) with a reduction in the [^18^F]FET signal occurring at day 6 (0.29 ± 0.13; *p* ≤ 0.01) (**Figure S3B**). TMZ treatment did not affect the [^18^F]DPA-714 T/B ratios and this signal remained stable over time.

Within the TMZ group, the treatment resulted in reduced [^18^F]FET-derived tumor volumes (0.008 ± 0.003 cm^3^; *p* ≤0.05), compared to DMSO-treated animals (0.02 ± 0.004 cm^3^; *p* ≤ 0.001). Also, at day 6, the [^18^F]FET-derived tumor volumes were also significantly reduced following TMZ treatment (0.023 ± 0.003 cm^3^; *p* ≤ 0.0001) (**Figure 2B**). Volumetric analysis of [^18^F]DPA-714 in the tumor region showed tracer uptake increase in DMSO-treated group (0.027 ± 0.005 cm^3^; *p* ≤ 0.0001), and significantly lower volumes after TMZ therapy at the same time point (0.018 ± 0.004 cm^3^; *p* ≤ 0.001) (**Figure 2C**). These findings were further validated by the comparison of [^18^F]DPA-714 PET/MRI with TSPO IHC, showing spatial agreement of tracer uptake with histology (**Figure S4**).

### Volumetric analyses show increased unique uptake of [^18^F]DPA-714 after therapy

**Figure 3:**
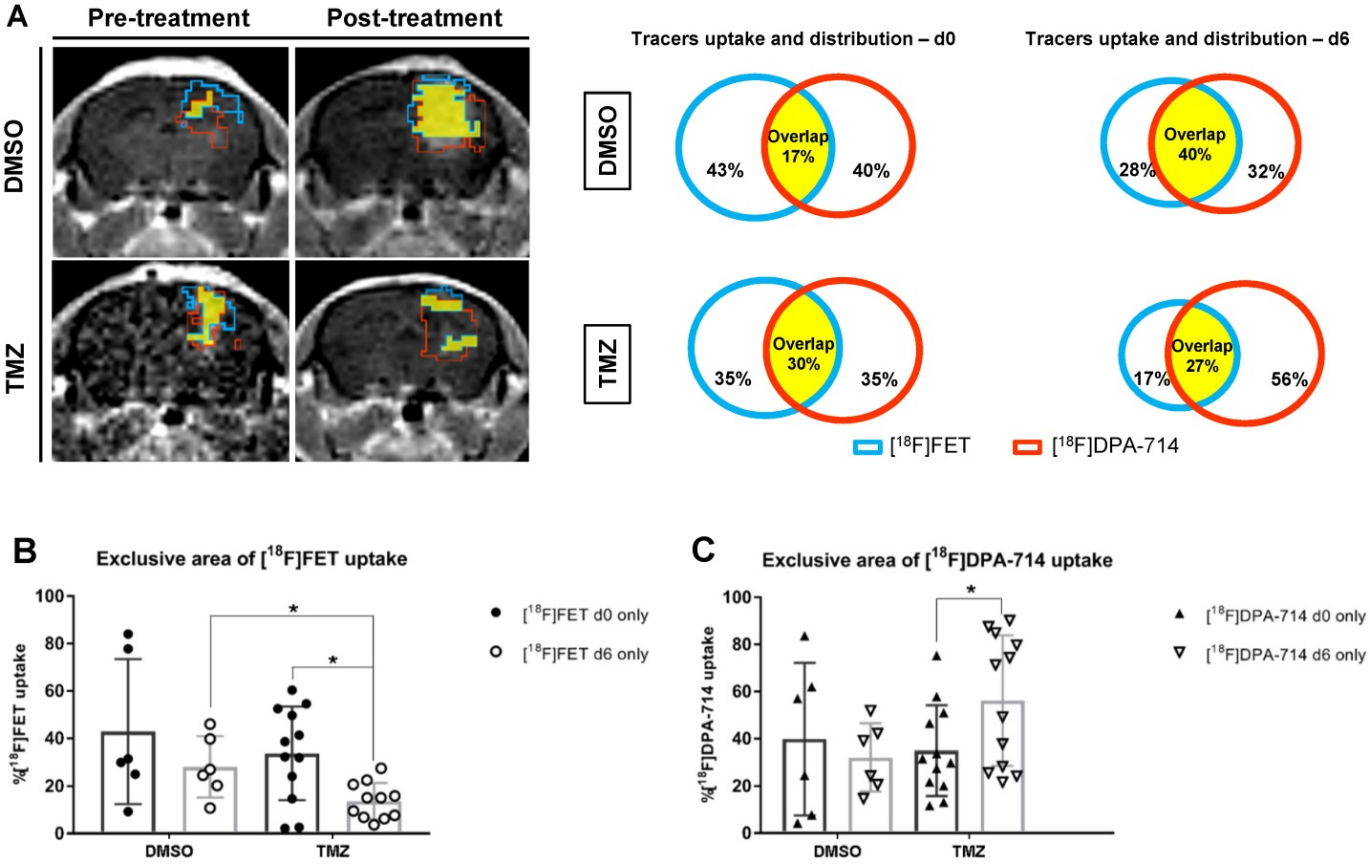
** Volumetric analysis defines the distribution of tracers in the tumor microenvironment and highlight specific therapy-induced alterations in the uptake of individual tracers. (A)** Representative contrast-enhanced MR T1w images and correspondent quantitative analysis of single tracers uptake and total distribution pre-and post-treatment in DMSO -and TMZ treated groups; the green lines represent [^18^F]FET unique area, red lines represent [^18^F]DPA-714 unique area and the yellow filled space is the overlap of both tracers. **(B)** [^18^F]FET and **(C)** [^18^F]DPA 714 tracer uptake volumes in DMSO- and TMZ-treated groups. Differences were tested for significance using a paired t-Test and Wilcoxon test with Bonferroni correction. DMSO: dimethyl sulfoxide; TMZ: temozolomide.

The quantitative volumetric analysis of exclusive areas of [^18^F]DPA-714 and [^18^F]FET uptake, as well as regions of tracer overlap, were performed (**Figure 3A - MRI images**). The tracer overlap increased from 17% to 40% after 6 days in DMSO control. On the contrary, the area of tracers overlap remained fairly stable at about 30% following TMZ treatment (**Figure 3A - diagrams**). Moreover, [^18^F]FET uptake was reduced by day 6 in TMZ-treated mice (14.37 ± 9.2 %; *p* ≤ 0.05). Comparison of day 6 between TMZ- and DMSO-treated groups also showed a significant reduction of [^18^F]FET uptake (20.05 ± 7.6 %; *p* ≤ 0.05) (**Figure 3B**). Interestingly, the same analysis demonstrated the opposite effect with unique [^18^F]DPA-714 tracer uptake: an increase in uptake following TMZ treatment (21.22 ± 9.8 %; *p* ≤ 0.05) with stability in DMSO controls (**Figure 3C**). Analyses of the individual mice in the TMZ-treated group were classified as responders and non-responders based upon the relative percentage of tumor volume change where the responders group included tumor volumes reduced by more than 20%. With this criteria, three responder mice demonstrated a positive correlation between tumor volume reduction to a reduction of [^18^F]DPA-714 uptake (**Figure S5A, #3 #8 and #10**). In 5 animals, 2 responders (**Figure S5A, #4 and #5**) and 3 non responders, in which the tumor reduction was between 9.9% and 14.5% (**Figure S5A, #2 #11 and #12**), the uptake of [^18^F]DPA-714 was increased or remained stable post therapy. The analyses of T/B ratios relative to [^18^F]DPA-714 and of the exclusive [^18^F]DPA-714 uptake showed heterogeneous levels among the cases and no statistical difference was detected between responders and non-responders pre- and post-therapy (**Figure S5B-C**).

### TMZ therapy does not change TSPO protein levels

To further investigate potential reasons for the increase in exclusive [^18^F]DPA-714 areas after TMZ treatment, protein expression levels of TSPO and Iba-1 in DMSO- and TMZ-treated animals were analyzed (**Figure S6A**). The higher tumor volumes in the DMSO-treated animals may suggest higher TSPO levels in the right hemisphere of these mice, compared to the TMZ-treated group, because tumor cells express TSPO as well. However, after protein normalization, no significant differences were detected for Iba-1 and TSPO, in the DMSO- and TMZ-treated group in the ipsi (L)-or contra (R)-lateral hemisphere between the two groups (**Figure S5B**).

### TSPO is increased in peri-tumoral tissues in response to TMZ and is associated with Iba1^+^ GAMMs and astrocytes

**Figure 4:**
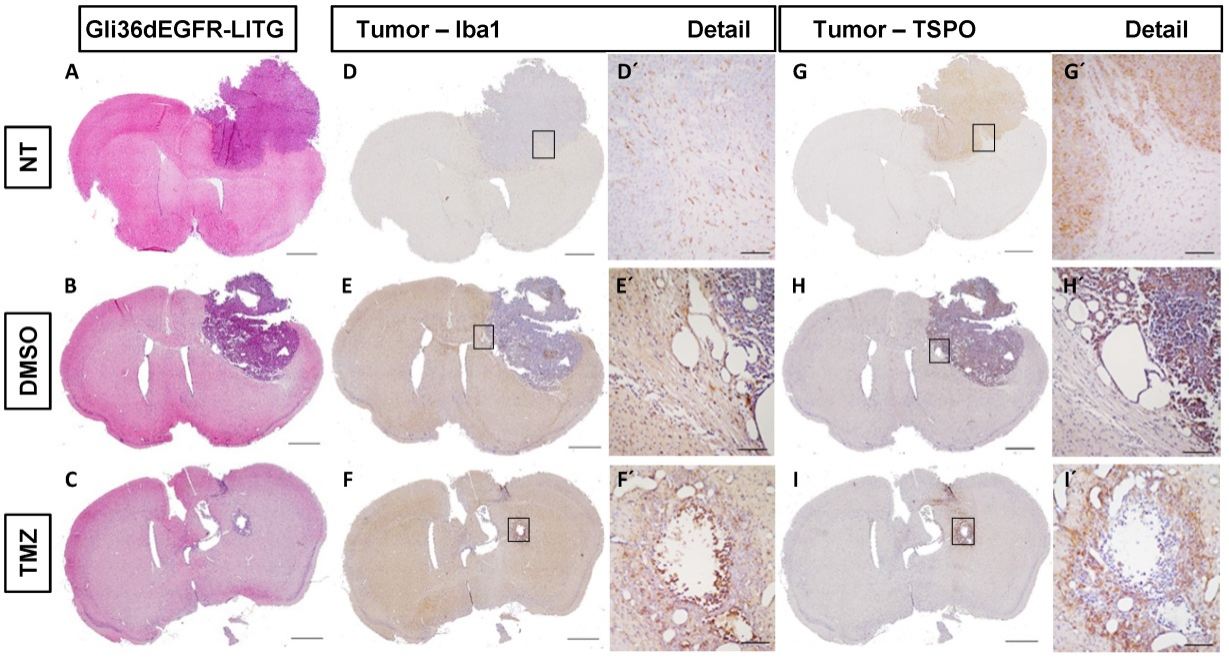
** Histological analysis of human glioma cells (Gli36dEGFR-LITG) orthotopically implanted in NMRI^nu/nu^ mice revealed the presence of reactive GAMMs infiltrating the tumor in controls and DMSO-treated animals, and within the tumor in the TMZ-treated group. On the contrary, TSPO signal mainly originated from the tumor in controls and DMSO-treated groups, while in TMZ-treated animals it was located at the periphery of the remained tumor mass.** Hematoxylin eosin staining **(A-C)** and immunohistochemistry for Iba1 **(D-F)** and TSPO **(G-I)** performed in histological sections of brains harvested from tumor bearing mice not treated (NT - upper lane), treated with vehicle (DMSO - middle lane) and treated with temozolomide (TMZ - lower lane), scale bar 1000 µm. The black squares represent details of Iba1 **(D´-F´)** and TSPO **(G´-L´)** and are displayed in a 10x magnification, scale bar 100µm. DMSO: dimethyl sulfoxide; GAMMs: gliomas-associated microglia/macrophages; NT: non-treated; TMZ: temozolomide.

**Figure 5:**
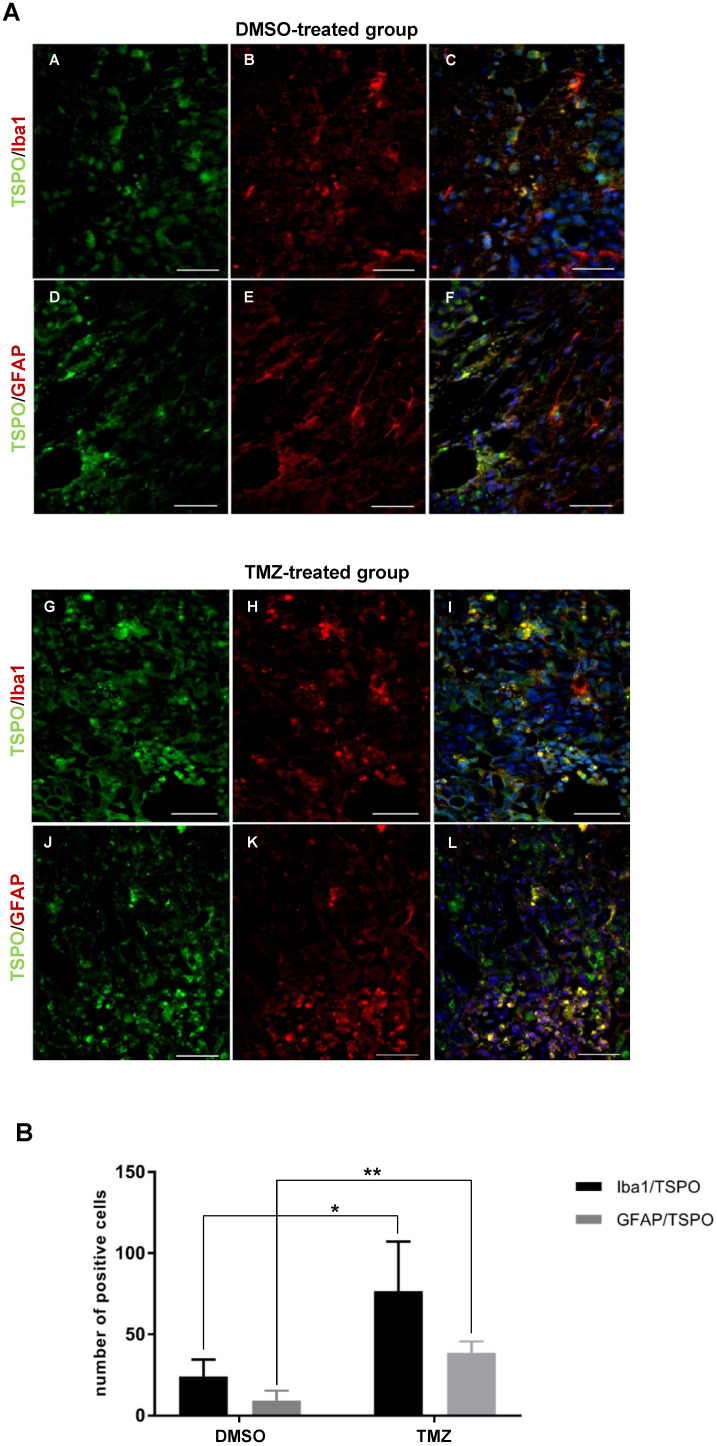
** The TME of animals treated with temozolomide showed the presence of activated GAMMs and astrocytes expressing TSPO. (A)** Paraffin embedded tumor sections labeled for TSPO **(green - A, D)** and Iba1 **(red - B, E)** or TSPO **(green - G, J)** and GFAP **(red - H, K); C, F, I, L** represent merged images; 40x magnification, scale bar 100µm. In blue the nuclear staining with DAPI (scale bar 50 µm). The dotted line separates the tumor area (marked with the letter T) from the peri-tumoral area. **(B)** Quantification of the number of activated GAMMs and astrocytes in biological triplicates. Differences were tested for significance using unpaired t-test. DMSO: dimethyl sulfoxide; GAMMs: gliomas-associated microglia/macrophages; GFAP: glial fibrillary acidic protein; Iba1: ionized calcium-binding adapter molecule 1; TMZ: temozolomide; TSPO: translocator protein.

To examine therapy-induced changes in the TME, IHC was performed on tissues obtained from non-treated, DMSO- and TMZ-treated mice with H&E- and specific Iba1-, TSPO- and GFAP-staining. The H&E staining validated the efficacy of TMZ treatment in reducing tumor volumes compared to both controls (**Figure 4A-C**). Iba1 staining revealed the presence of microglia cells surrounding and infiltrating the tumor tissue in the entire right hemisphere of the non-treated tumor-bearing mice (**Figure 4D-D'**). On the contrary, microglia cells were only detected in the peri-tumoral tissues or in few clusters within the tumor mass in the DMSO- and TMZ-treated animals (**Figure 4E-F'**). In the two control groups, tumor cells and perivascular cells were positive for TSPO within the tumor mass (**Figure 4G-H' and Figure S7B-D''**). On the other hand, TSPO positive cells were found only in the area surrounding the remaining glioma tissue post TMZ-treatment (**Figure 4I-I' and Figure S7F-F''**). Overall, GFAP IHC revealed the presence of GFAP expressing cells surrounding the tumor with a morphology typical of astrocytes. The GFAP positive area appeared to be separated and complementary to the TSPO expressing tumor area in both of the control groups (**Figure S7A-A'', C-C''**). GFAP expressing cells were also present within the remaining glioma tissue and in proximity of vessels in the TMZ-treated mice (**Figure S7E-E''**). These findings were further confirmed by immunofluorescence analysis performed to investigate the presence of activated microglia cells and astrocytes within the TME. Microglia cells and astrocytes localized primarily at the periphery of the tumor in DMSO-treated cells with little co-localization of TSPO and GFAP (**Figure 5A,B,F**). Further, microglia cells were found in clusters within the tumor tissue and reactive astrocytes were identified at the edge of and invading the tumor mass post TMZ treatment (**Figure 5A,G-L**). Lastly, TMZ treatment yields a significant increase in co-localization of TSPO/Iba1 and GFAP/TSPO (mean diff. ± SE: 52.6 ± 18.6; *p* ≤ 0.05 and 29.3 ± 5.34; *p* ≤ 0.001, respectively) (**Figure 5B**).

### Effect of TMZ therapy on infiltrating myeloid-derived cells

**Figure 6:**
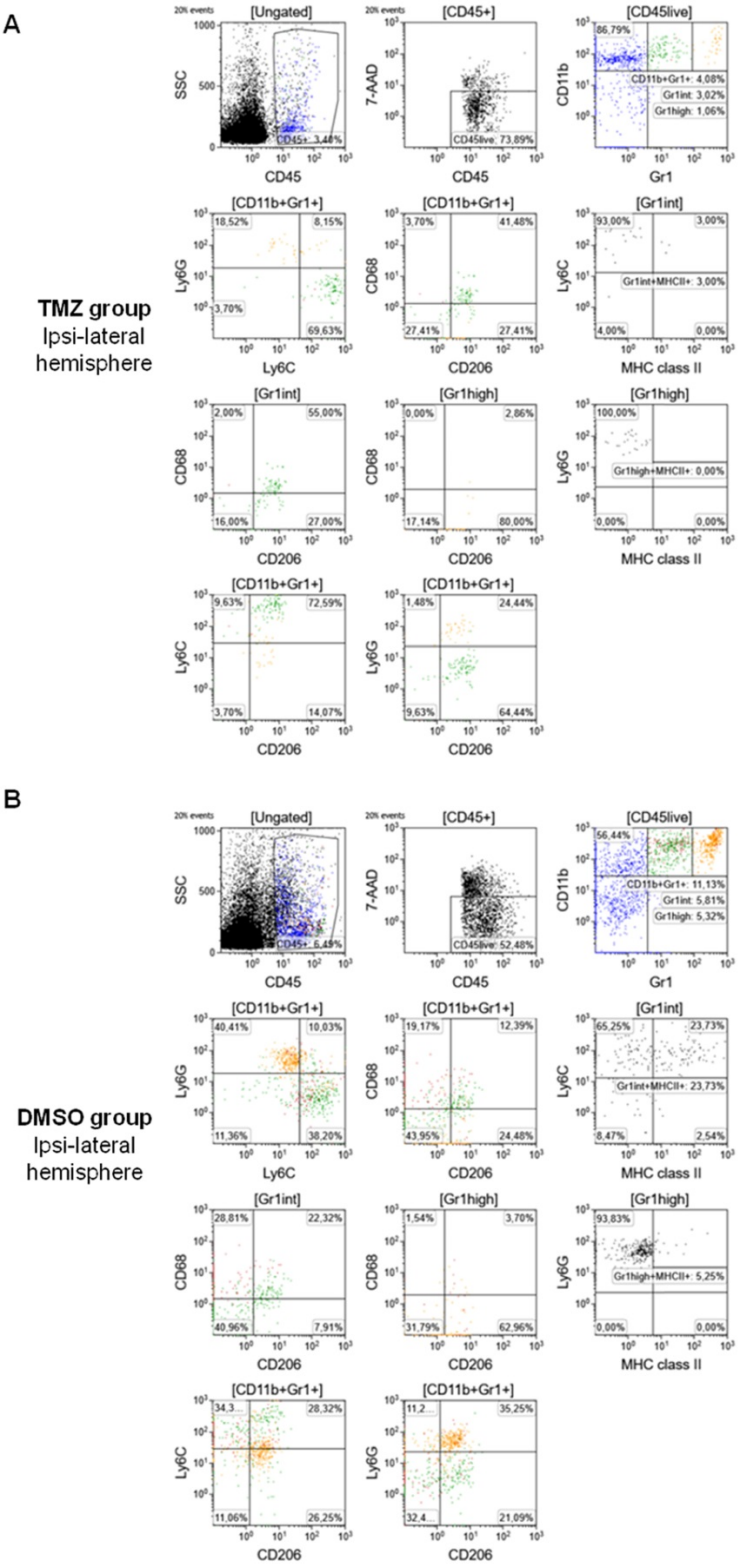
** Flow cytometry characterization of the TME identified high expression of M2 markers (CD68^+^CD206^+^) and Mo-MDSCs (CD11b^+^Gr1^+^Ly6G^-^Ly6C^+^) in TMZ-treated animals.** Cell sorting and gating protocol adopted for the analysis of **(A)** TMZ-treated mice and **(B)** DMSO-treated mice. DMSO: dimethyl sulfoxide; GFAP: glial fibrillary acidic protein; Iba1: ionized calcium-binding adapter molecule 1; TMZ: temozolomide; TSPO: translocator protein.

To further evaluate the phenotype of tumor-infiltrating cells and the therapy-induced changes in the TME, multiparametric flow cytometry was performed as described in the materials and methods section (**Figure 6**). TMZ treatment reduced the frequency of total MDSCs compared to the DMSO-treated group (CD11b^+^Gr1^+^: 4.08% and 11.13%, respectively) and produced a difference in the MDSCs phenotype. Specifically, in the TMZ-treated animals, a prevalence of Mo-MDSCs was detected in the TME (CD11b^+^Gr1^+^Ly6G^-^Ly6C^+^: 69.63%). TMZ treatment increased surface expression of CD68 and CD206, markers of an M2-like phenotype (CD11b^+^Gr1^+^CD68^+^CD206^+^: 41.48%) mostly in the Mo-MDSCs population (Gr1^int^CD68^+^CD206^+^: 55% and CD11b^+^Gr1^+^Ly6C^+^CD206^+^: 72.59%) (**Figure 6A**). There was no significant difference in controls: MDSCs were equally identified as PMN-MDSCs (CD11b^+^Gr1^+^Ly6G^+^Ly6C^-^: 40.41%) and Mo-MDSCs (CD11b^+^Gr1^+^Ly6G^-^Ly6C^+^: 38.2%). There was less expression of M2 markers relative to the TMZ-treated group (CD11b^+^Gr1^+^CD68^+^CD206^+^: 12.39%) with no differences between the MDSCs populations (Gr1^int^CD68^+^CD206^+^: 22.23%; CD11b^+^Gr1^+^Ly6C^+^CD206^+^: 28.32%; CD11b^+^Gr1^+^Ly6G^+^CD206^+^: 35.25%) in DMSO-treated animals (**Figure 6B**). Furthermore, TMZ treatment reduced the expression levels of MHC class II, a marker of activation and differentiation of Mo-MDSCs, relative to the DMSO group (Gr1^int^Ly6C^+^MHC II^+^: 3% and 23.73%, respectively).

## Discussion

The aim of this study was to assess the suitability of a multi-tracer PET/MRI combination to investigate the dynamic changes in the TME in response to TMZ therapy in a pre-clinical gliomas model. In this study, we demonstrate that the combination of [^18^F]FET and [^18^F]DPA-714 (TSPO) PET allows to monitor TMZ-induced changes in the TME and to identify specific reactive areas of immune cell infiltration. Moreover, we were able to spatially visualize the therapy-induced modifications of the TME non-invasively. Unchanged [^18^F]DPA-714-PET uptake after TMZ may be explained by peri-tumoral GAMMs and astrocytes expressing TSPO as well as by the infiltration of myeloid-derived cells expressing TSPO.

Despite all scientific effort in the field, the development of novel treatments for gliomas remains challenging. During the last decade, immunotherapy gained an important role in this landscape and has risen great expectations over the years 33-35. Glioma-associated microglia/macrophages (GAMMs), together with other myeloid-derived cells such as dendritic cells, neutrophils and myeloid-derived suppressor cells (MDSCs), promote tumor progression and the establishment of an immunosuppressive tumor microenvironment 6,36. MDSCs in particular are emerging as important immunosuppressive players, inhibiting T-cells and NK cells, promoting Treg expansion and suppressing pro-inflammatory responses in different disease models like traumatic brain injury (TBI) and brain cancer 37,38. The high heterogeneity of the TME plays a central role in drug resistance and treatment failures 39. In addition, the lack of biomarkers, especially for the tumor microenvironment and the assessment of immunological response, makes it difficult to monitor treatment outcomes beyond morphological volumetric assessment with MRI 40. More specific, non-invasive imagine techniques are therefore essential, both as diagnostic and follow-up tools, allowing clinicians to take prompt decisions 41,42.

O-(2-[^18^F]fluoroethyl)-L-thyrosine ([^18^F]FET) is a PET tracer routinely used for diagnosis of brain tumors, image-guided surgical resection 43, treatment planning and response in glioma 44-46. [^18^F]FET uptake is mediated by the system L amino acid transporter (LAT1) 47. Cancer tissue hijack and alter metabolic pathways to increase amino acid transport for cell proliferation and survival 48,49. Therefore, [^18^F]FET has been utilized as an imaging biomarker targeting tumor cells and tumor progression 50,51.

We confirmed that [^18^F]FET-PET, together with CE-MRI, can be used for monitoring glioma growth and TMZ-treatment response. However, [^18^F]FET PET/MRI does not provide information on reactive changes in the immunosuppressive myeloid cell compartment in gliomas upon clinical routine TMZ therapy. A few studies reported that LAT1 is overexpressed and essential for the differentiation of T-cells but it is not known if the uptake of [^18^F]FET is influenced by this immunological component 52,53. However, approaches combining [^18^F]DPA-714-PET with other PET tracers or imaging modalities demonstrated promising results in the characterization of the TME in preclinical glioma models 22, as well as in glioma patients 23.

We and others showed that [^18^F]DPA-714 supports the identification of unique TSPO-expressing regions of the TME 20,22 that may represent areas with immunosuppressive myeloid cells 23 and glioma infiltration 24,54. In spite of these promising results, very few studies describe the suitability of TSPO-binding radioligands for the monitoring of therapy outcomes 55,56 and even fewer specific for [^18^F]DPA-714 in glioma. Awde and colleagues showed the efficacy of [^18^F]DPA-714 in monitoring the disease progression and response in a rat model of glioma treated with ErPC3 (alkylphosphocholine erufosine) 57. None of the studies investigated the role of [^18^F]DPA-714-PET in conjunction with [^18^F]FET-PET to monitor TMZ-induced changes in gliomas.

In our study, we have demonstrated the utility of combining [^18^F]FET and [^18^F]DPA-714-PET to monitor TMZ-therapy response, as evidenced in the volumetric analysis of [^18^F]FET and [^18^F]DPA-714 uptake within the tumor mass.

As expected, [^18^F]DPA-714-PET uptake was significantly reduced after 6 days of TMZ treatment, compared to DMSO-treated group measured at the same time point. Unchanged TSPO-PET uptake post-TMZ may be explained by peri-tumoral GAMMs and astrocytes expressing TSPO as well as by the infiltration of myeloid-derived cells expressing TSPO. Considering that tumor cells also express TSPO, these results have a positive correlation with reduction in tumor volumes, as seen by contrast-enhanced (CE)-MRI which is in line with the decrease of [^18^F]FET volumes. Interestingly, analysis of the T/B ratios revealed a stable [^18^F]DPA-714 uptake across all groups while TSPO levels of protein expression remained unchanged, as assessed by western blot. Moreover, the volumetric analysis of exclusive tracer uptake showed a significant increase of [^18^F]DPA-714 after TMZ therapy. One explanation could be a de-masking effect of peri-tumoral TSPO expression sources stemming from tumor shrinkage. This suggests that reactive immune cells present in the TME in response to chemotherapy may be an important denominator of TSPO expression. To further explore these potential mechanisms, tissue analysis with IHC and fluorescence labeling was performed. In line with stable TSPO levels in WB and increased [^18^F]DPA-714 uptake in exclusive areas of the TME, we found an increased number of peri-tumoral GAMMs and astrocytes expressing TSPO in TMZ-treated animals.

These findings are in accordance with other studies that investigated TSPO expression in glial cells. In a model of brain metastasis, *in vivo* TSPO-PET showed that not only microglia cells had increased TSPO expression, but also astrocytes 58. Furthermore, in a recent study Pannell and colleagues showed that TSPO is upregulated specifically in pro-inflammatory microglia/macrophages and astrocytes in a neuroinflammation model 59.

TMZ is known to have a myelosuppressive and anti-proliferative effect. TMZ treatment resulted in decreased MDSC infiltration in the TME, suggesting a prevalence of GAMMs or early-stage undifferentiated MDSC infiltration (observed as CD11b^+^Gr1^-^ cells in flow cytometry analysis) which may impact [^18^F]DPA-714 uptake. In addition, TMZ therapy reduced the expression levels of the MHC class II protein, a marker of activation and differentiation of Mo-MDSC into macrophages or dendritic cells, indicating a possible therapeutic effect on this pathway. Many studies reported the importance of targeting MDSCs and GAMMs in glioma, given the highly immunosuppressive nature of these cells 60-62. Various therapeutic approaches to target MDSCs in glioma have been developed with the aim to directly eliminate MDSCs or inhibiting the migration and expansion into the TME 63. Many of these strategies consist in combination therapies using chemotherapeutic agents like TMZ and immunotherapy 63,64. However, the effect of TMZ on MDSCs has not been largely investigated, especially in patients, and further studies are needed. Our group previously reported a correlation between [^18^F]DPA-714 uptake and frequency of myeloid-derived infiltrating cells in glioma patients 23. In this study, we expanded upon that observation to show cells infiltrating the TME were expressing markers of a M2-like phenotype following TMZ therapy. These infiltrating cells may represent a population of therapy-resistant cells and further investigations will be performed to better define the correlation between TSPO expression and the cellular phenotypes. Therefore, the addition of [^18^F]DPA-714 in the diagnostic work up of glioma patients may support the identification of areas of tumor, infiltration, therapy resistance and may even support the selection and stratification of patients eligible for novel immunotherapies. Larger scale clinical studies will be needed to further clarify the potential of [^18^F]DPA-714 in glioma patient management and to understand the prognostic value of a multi-modality imaging paradigm. A step in this direction was made by Ina Ly and colleagues using functional MRI to investigate therapy-induced changes in tumor physiology and the vascular state in newly diagnosed GBM patients treated with radiation therapy and TMZ. The aim of the study was to identify a better therapeutic window and to distinguish between early responders and non-responders 65.

In conclusion, our study indicates that [^18^F]FET in conjunction with contrast enhanced MRI is a suitable tool for monitoring TMZ therapy-response *in vivo*, while [^18^F]DPA-714 may serve as a complementary molecular imaging marker to image specific immune cell infiltration and reactive changes in the TME.

As shown by the unexpected increase of exclusive areas of [^18^F]DPA-714 uptake, the anti-proliferative effect of TMZ therapy induced an increase in immunoreactivity of TSPO-expressing glial cells surrounding the tumor mass. These findings were further confirmed by histological analyses showing that tumor cells expressed TSPO but not GFAP. This observation is an important step to aid in discrimination of peri-tumoral TSPO sources. Further analyses are needed to discriminate the specific cell populations that express TSPO within the TME, such as perivascular cells and the subpopulations of tumor-associated infiltrating cells. Multi-parametric flow cytometry helped to define the phenotype of infiltrating GAMMs and MDSCs and these findings might be of help to select the best treatment approach and improve the efficacy of combination therapies in glioma, e.g. dexamethasone and check-point inhibitors. A better knowledge of the TSPO expression sources and the underlying TSPO dynamics upon therapy will be crucial in order to improve our understanding of *in vivo* TSPO-targeted imaging in gliomas. For instance, endothelial binding and neoangiogenesis may affect TSPO levels and tracer uptake, contributing to the therapy outcomes. The use of [^18^F]DPA-714 in combination with other tracers, specific for myeloid-derived cells as ^89^Zr-labeled anti-CD11b Ab for example 16, and a better characterization of TSPO-expressing endothelial cells might be useful in this regard. Furthermore, given the limitations due to the use of (i) nude mice without a fully competent immune system, (ii) cell lines which do not reflect entirely the heterogeneity of the tumor tissue, and (iii) the mechanical damage derived by the intracranial injection of tumor cells, which might lead to extracranial tumor growth and influence immune cell infiltration in the TME, future studies should focus on using e.g. immunocompetent, syngeneic mouse models. However, these findings have significant relevance for the use of TSPO-targeted PET imaging, since [^18^F]DPA-714 could be a useful imaging biomarker for monitoring reactive cell infiltration. Finally, TSPO might be taken into consideration (i) for developing personalized and targeted therapies and (ii) to identify novel options for immunotherapy for patients with glioma.

## Supplementary Material

Supplementary figures.

## Abbreviations

CCL2: C-C motif chemokine ligand 2
CE: contrast-enhanced
CNS: central nervous system
CSF1: colony stimulating factor 1
CT: computer tomography
DAPI: 4′,6-diamidino-2-phenylindole
DMEM: Dulbecco's modified eagle medium
DMSO: dimethyl sulfoxide
[^18^F]DPA-714: *N,N*-diethyl-2-(2-(4-(2-[^18^F]fluoroethoxy)phenyl) 5,7-dimethylpyrazolo[1,5-a]pyrimidin-3-yl)acetamide
ErPC3: alkylphosphocholine erufosine
FCS: fetal calf serum
[^18^F]FET: O-(2-[^18^F]fluoroethyl)-L-thyrosine
GAMMs: gliomas-associated microglia/macrophages
GAPDH: glyceraldehyde-3-phosphate dehydrogenase
GBM: Glioblastoma multiforme
Gd: gadolinium
GFAP: glial fibrillary acidic protein
IHC: immunohistochemistry
Iba1: ionized calcium-binding adapter molecule 1
IL-4: interleukin 4
i.v.: intravenous
MBq: megabecquerel
MDSCs: myeloid-derived suppressor cells
Mo-MDSCs: monocytic myeloid-derived suppressor cells
MRI: magnetic resonance imaging
PET: positron emission tomography
PFA: paraformaldehyde
p.i.: post-implantation
PMN-MDSCs: polymononuclear myeloid-derived suppressor cells
PMSF: phenylmethylsulfonyl fluoride
PVDF: polyvinylidene difluoride
T/B: tumor-to-background
T2w/T1w: T2 weighted/T1 weighted
TGFβ: transforming growth factor β
TME: tumor microenvironment
TMZ: temozolomide
TSPO: translocator protein
VOI: volume-of-interest
