# Supplementary material for: Imaging temozolomide-induced changes in the myeloid glioma microenvironment
Source: Theranostics. 2021 Jan 1;11(5):2020–33. doi: 10.7150/thno.47269 (PMC7797694; doi:10.7150/thno.47269)
Supplement: Supplementary file 1 — Supplementary figures. [file thnov11p2020s1.pdf]

Supplementary Figures

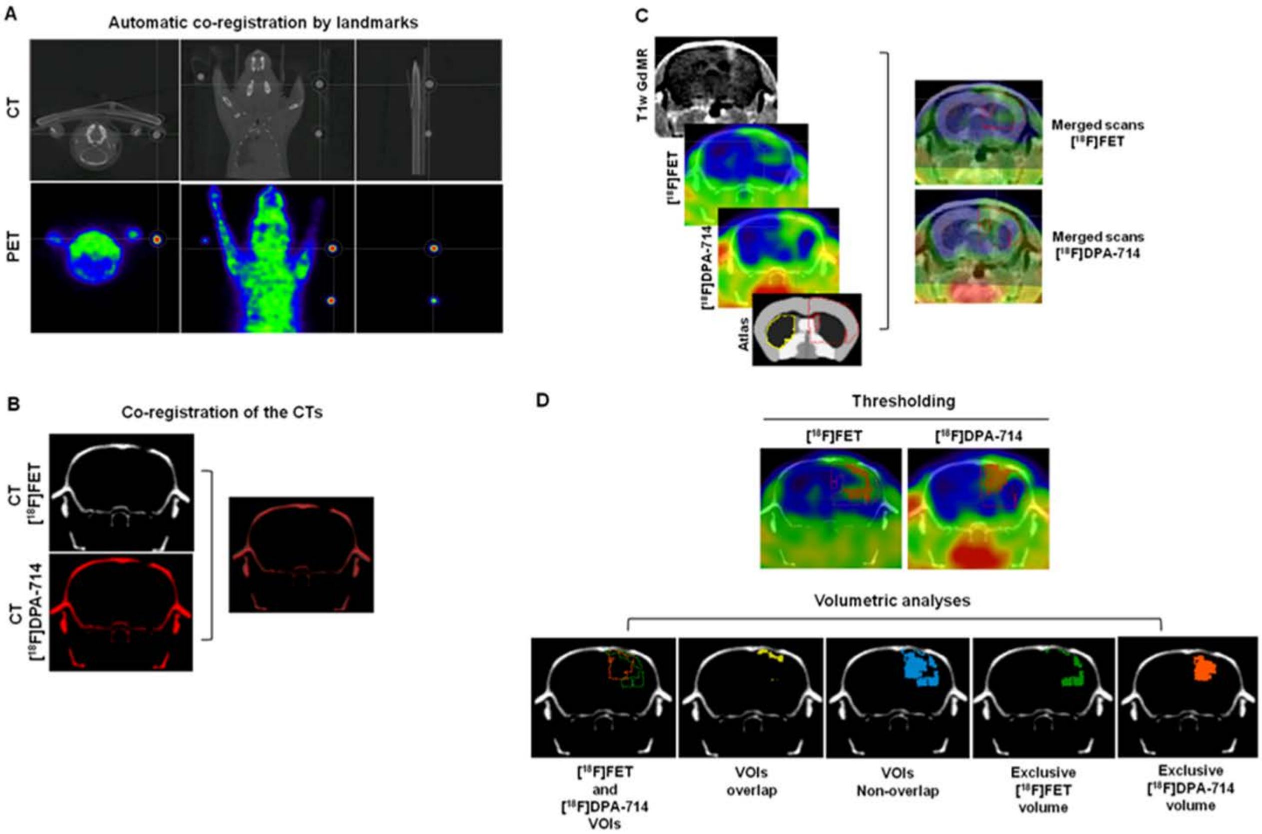

Supplementary Figure S1. Workflow representing the different steps of the data analysis process. Images co-registration and volumetric analysis approach. **(A)** Representative image of the automatic co-registration of CT and PET images using landmarks (circles) as reference. **(B)** Representative image of the co-registration of the two CTs obtained during [^{18}F]FET-PET and [^{18}F]DPA-714-PET imaging. **(C)** Representative workflow for the co-registration of T1w Gd-MRI with [^{18}F]FET-PET, [^{18}F]DPA-714-PET and the atlas with the two VOIs. **(D)** Representative images showing the thresholding of the right hemisphere VOI for [^{18}F]FET-and [^{18}F]DPA-714, and the volumetric analyses approach.

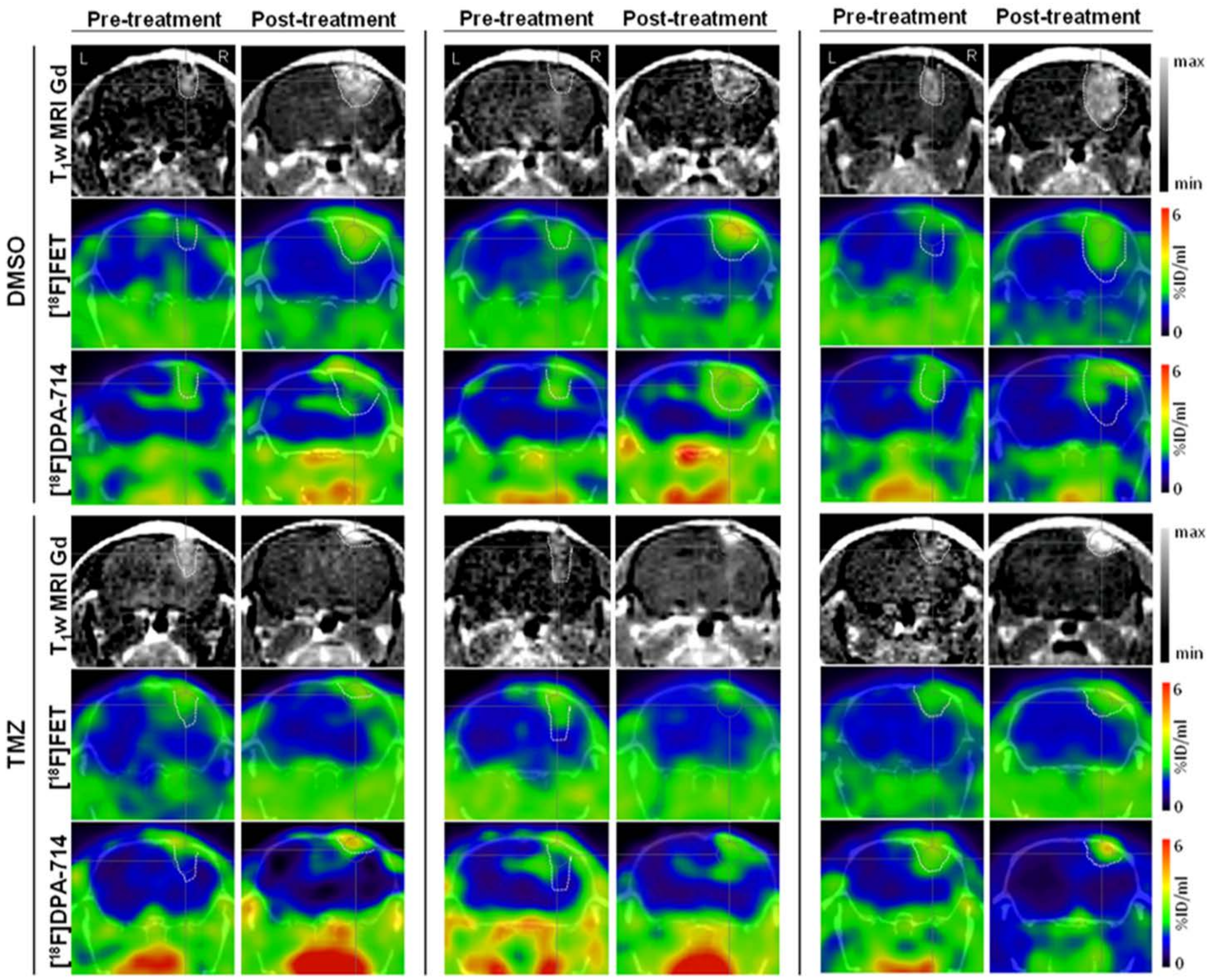

Supplementary Figure S2. Additional multimodal dual-tracer PET/MR images. Representative transaxial T_1wMRI Gd and PET images for [^{18}F]FET and [^{18}F]DPA-714 (top to bottom) fused with CT of control (DMSO) and TMZ-treated animals, pre- and post-treatment (left to right). The dotted line indicates the tumor area depicted by MRI and transferred to PET images. L and R indicate left- and right-hemisphere. DMSO: dimethyl sulfoxide; TMZ: temozolomide.

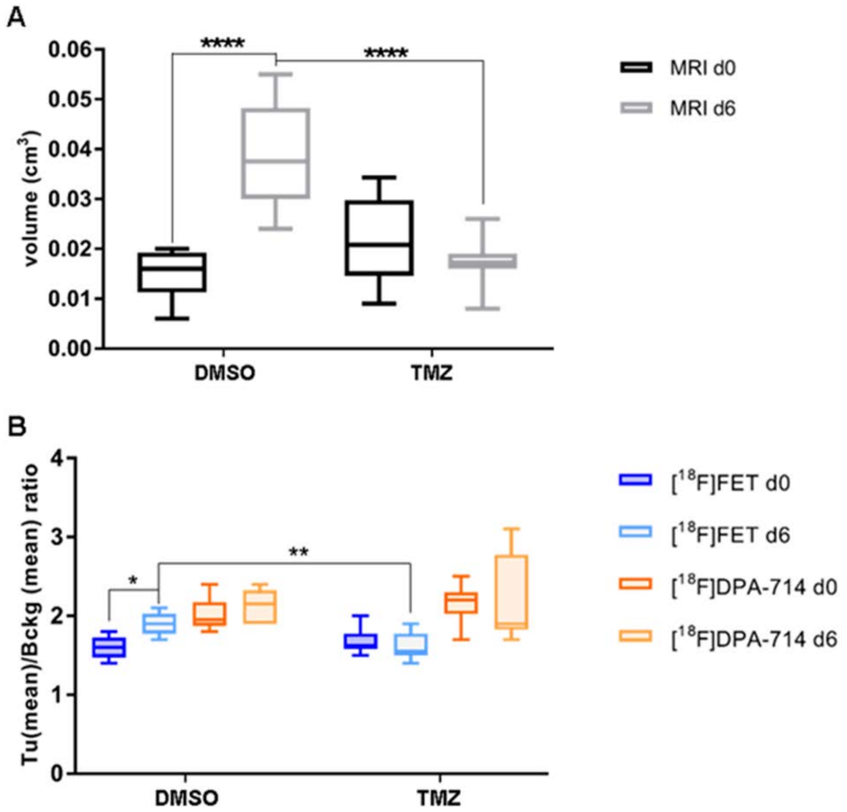

Supplementary Figure S3. Quantification of tumor volume based on T1w CE-MRI and Tu(mean)/Bckg(mean) ratio. Volumetric analyses showed increased tumor volume and [^{18}F]FET uptake in the DMSO group, and a reduction of tumor volume and [^{18}F]FET uptake after TMZ treatment, comparing TMZ- and DMSO-treated animals at day 6. **(A)** Quantitative analysis of the tumor volume (cm^3/ml) based on T1 MRI with contrast agent (Gadovist) images, pre- and post-treatment. **(B)** Quantitative analysis of [^{18}F]FET and [^{18}F]DPA-714 T (mean)/B (mean) uptake ratio after 6 days from the beginning of the therapy with DMSO (vehicle) and 50 mg/kg TMZ. Differences intra- and inter-groups were tested for significance using paired t-Test, Wilcoxon test and Mann-Whitney test with Bonferroni correction.

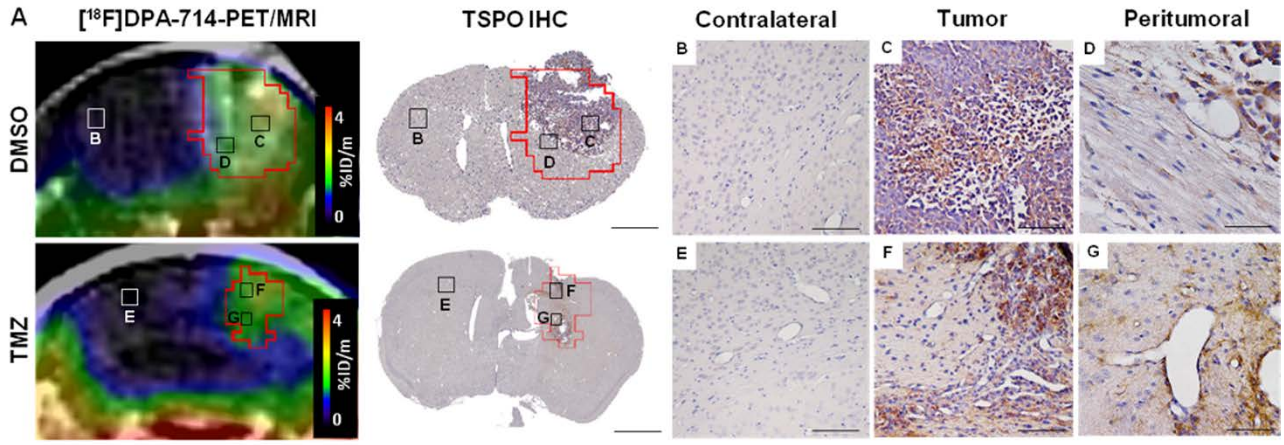

Supplementary Figure S4. Comparison of [^{18}F]DPA-714 with TSPO histology. Co-registration of PET/MRI images and TSPO IHC showed good spatial agreement. **(A)** Comparison of [^{18}F]DPA-714 PET/MRI images with immunochemistry for TSPO (scale bar 1000 µm). **(B-G)** Details at higher magnification showing absence of TSPO in the contralateral hemisphere and TSPO reactivity within the tumor tissue (scale bar 100 µm – Contralateral and Tumor - and 50 µm - Peritumoral). To be noticed that the squares representing D (peritumoral area) and E (contralateral side) are placed taking in account the tissue deformation resulting from the alcohol de-hydration process of the histology preparation and for this reason are slightly moved in the TSPO IHC image compared to the PET/MRI image.

A

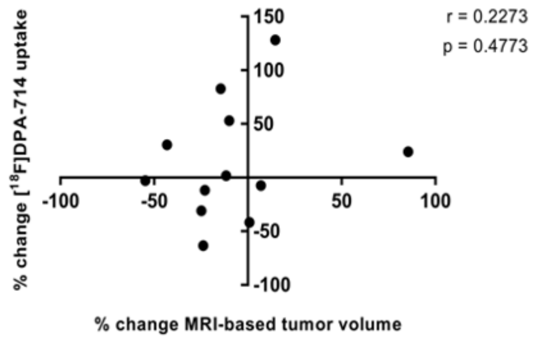

	% change MRI-based tumor volume	% change [18F]DPA-714 uptake
1	7	-7,32
2	-9,9	53
3	-23,8	-63,4
4	-54,7	-2,8
5	-43,2	30,5
6	85,5	24,1
7	14,6	128,2
8	-24,8	-31
9	0,8	-41,7
10	-22,9	-11,8
11	-11,6	1,7
12	-14,5	82,7

B

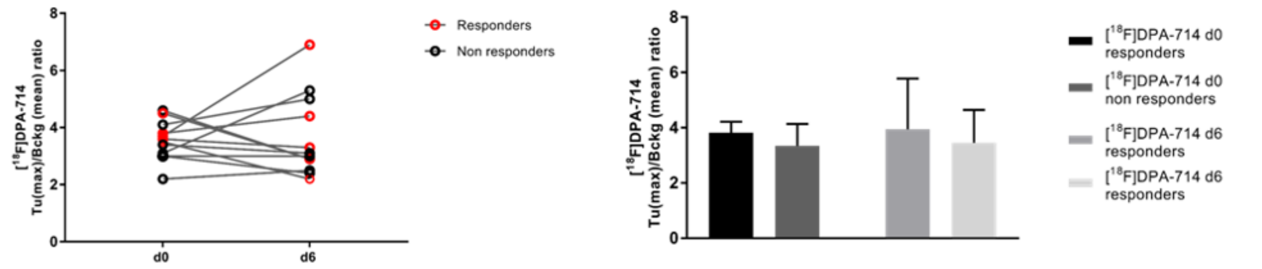

C

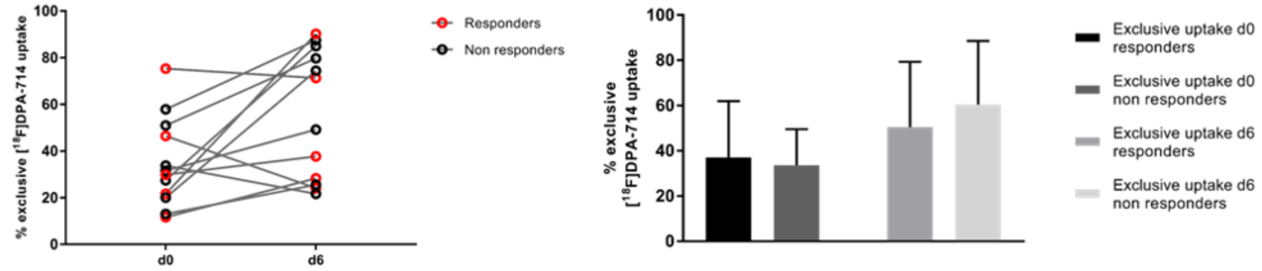

Supplementary Figure S5. Case by case analysis of "responders" and "non-responders" TMZ-treated mice showed no correlation between relative change of tumor volume and [^{18}F]DPA-714 uptake as well as no differences between responders and non-responders regarding [^{18}F]DPA-714 uptake pre-and post therapy. **(A)** Correlation of the percentage of MRI-based tumor volume change with the percentage change of [^{18}F]DPA-714 uptake. Single cases data are reported in the table on the right**. (B)** T/B ratios relative to [^{18}F]DPA-714 uptake pre- and post-treatment with TMZ in responder (red circles) and non responder cases (black circles). Data are also represented in bar graphs as mean ± SD. **(C)** Percentage of exclusive [^{18}F]DPA-714 uptake in TMZ-treated animals, responders (red circles) and non responders (black circles). Data are also represented in bar graphs as mean ± SD.

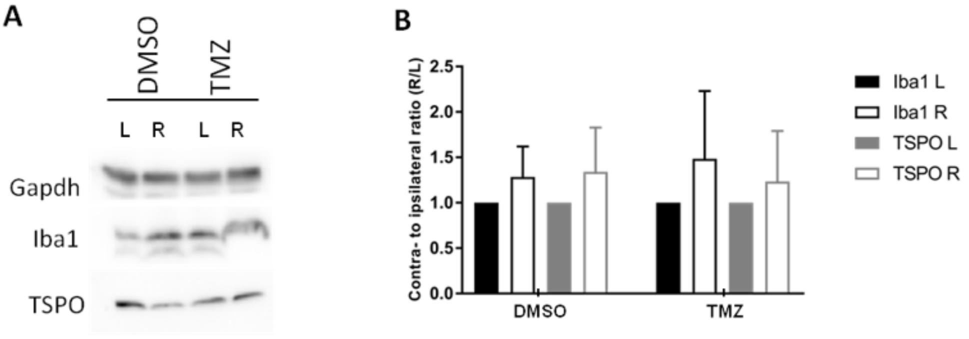

Supplementary Figure S6. Western blot analysis results. TMZ therapy did not significantly affect TSPO protein levels. **(A)** Representative western-blot for Iba1 and TSPO expression. **(B)** Quantification of the protein levels of Iba1 and TSPO in biological triplicates. All data are shown as ratio ipsi- to contralateral-side (R/L). R = right hemisphere; L = left hemisphere.

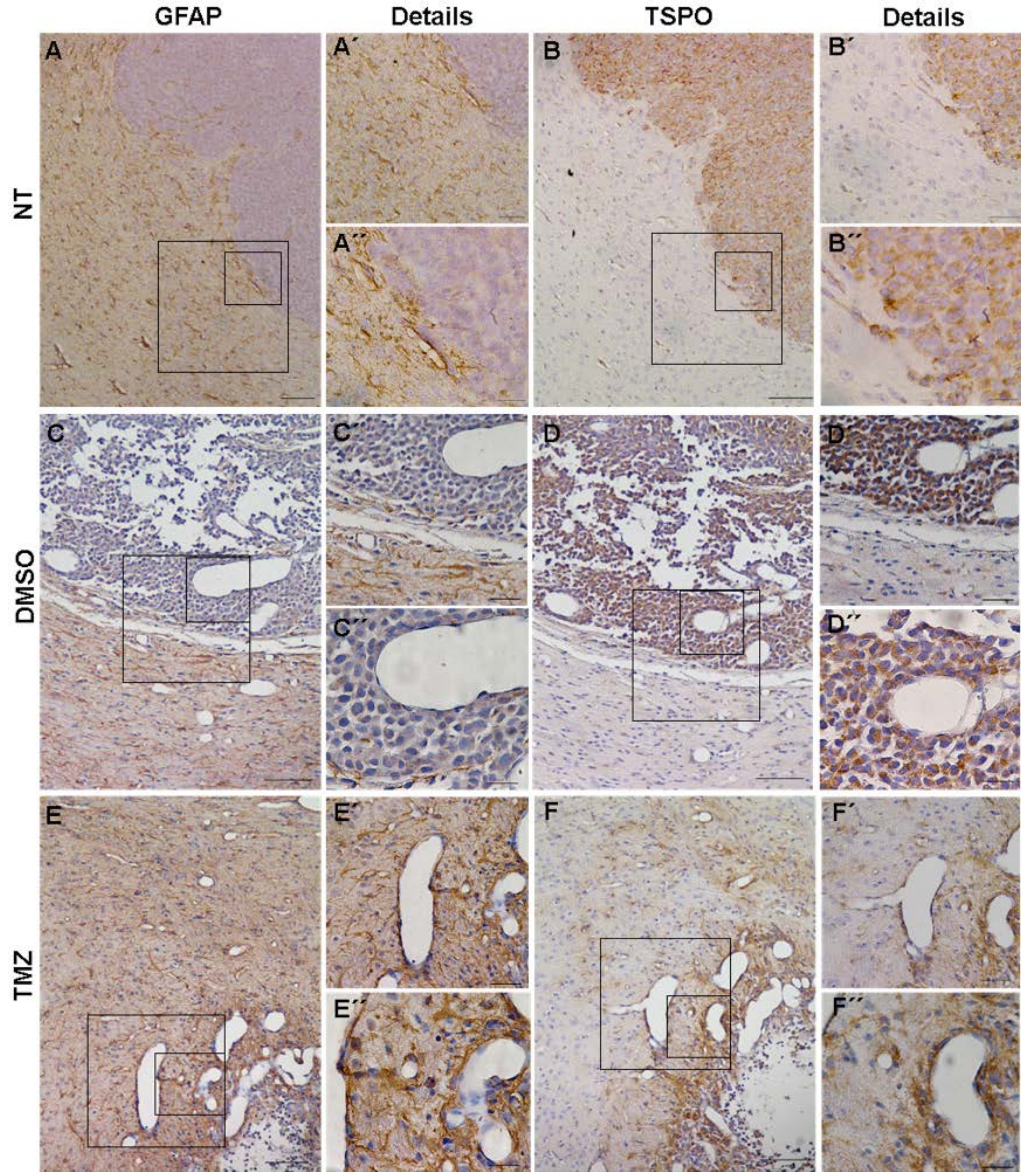

Supplementary Figure S7. Histological analysis of GFAP and TSPO in the TME. GFAP IHC revealed the presence of reactive astrocytes surrounding the tumor in controls and DMSO-treated animals, and within the tumor in the TMZ-treated group. TSPO expression resulted from tumor cells in non treated and DMSO-treated groups, while in TMZ-treated animals TSPO is located at the periphery of the remaining tumor mass. Immunohistochemistry for GFAP **(A - C - D)** and TSPO **(B - D - F)** performed in histological sections of brains harvested from tumor bearing mice not treated (upper lane), treated with vehicle (DMSO – middle lane) and treated with Temozolomide (TMZ – lower lane), scale bar 1000µm **(A – F)**. The bigger squares represent details of GFAP **(A´, C´, E´)** and TSPO **(B´, D´, F´)** and are displayed in a 20x magnification, scale bar 50µm. The smaller squares represent details of GFAP **(A´´, C´´, E´´)** and TSPO **(B´´, D´´. F´´)** in a 40x magnification, scale bar 50µm.
